# Aromatase Inhibitors and Risk of Metabolic and Cardiovascular Adverse Effects in Breast Cancer Patients—A Systematic Review and Meta-Analysis

**DOI:** 10.3390/jcm11113133

**Published:** 2022-05-31

**Authors:** Kamila Boszkiewicz, Agnieszka Piwowar, Paweł Petryszyn

**Affiliations:** 1 Department of Toxicology, Wroclaw Medical University, Borowska Street 211, 50-556 Wroclaw, Poland; agnieszka.piwowar@umw.edu.pl; 2 Department of Clinical Pharmacology, Wroclaw Medical University, Borowska Street 211a, 50-556 Wroclaw, Poland; ppetryszyn@wp.pl

**Keywords:** breast cancer, aromatase inhibitors, adverse effects

## Abstract

Aromatase inhibitors (AIs) have been considered first-line therapy for patients with hormone-dependent breast cancer due to their high efficacy and good tolerability. However, AIs are not free of adverse events, and studies show that therapy with AIs is associated with an increased risk of cardiovascular events and the development of insulin resistance and diabetes. We searched the Cochrane Central Register of Controlled Trials, PubMed and EMBASE up to 27 October 2020 for the prevalence of cardiovascular and/or metabolic adverse effects during treatment with AIs in postmenopausal women with breast cancer. A meta-analysis was performed using a random effects model. Odds ratios and 95% confidence intervals were calculated and illustrated using forest plot charts. We performed separate analyses depending on trial design. Twenty two studies met the inclusion criteria. AIs were associated with a higher risk of cardiovascular events, especially when we compared study arms in which AIs were used (alone or in sequence with TAM) with the arms in which TAM was used alone (OR = 1.16; 95%CI 1.04–1.30) or when comparing patients taking AIs alone to patients taking TAM alone or in sequence with AIs (OR = 1.24; 95%CI 1.11–1.38). A pooled analysis of five trials comparing adjuvant AIs to TAM showed the odds for arterial hypertension being 1.31 times higher for patients taking AIs; however, this did not reach statistical significance (OR = 1.31; 95%CI 0.47–3.65). We have not shown an increased risk of dyslipidemia or weight gain with the use of AIs. Our results suggest that postmenopausal women with breast cancer treated with AIs have an increased risk of cardiovascular events in comparison with TAM, potentially due more to a cardioprotective effect of the latter than the cardiotoxicity of AIs. We were unable to prove a similar association for hypertension, dyslipidemia, hyperglycemia or weight gain. Further high-quality RCTs and post-marketing safety observational studies are needed to definitively evaluate the impact of AIs on metabolic disorders in breast cancer patients.

## 1. Introduction

Breast cancer is the most common cancer in women, affecting 2.1 million women worldwide each year and causing the largest number of cancer-related deaths in women [1]. According to the data providing by American Cancer Society, 13% of women will develop breast cancer in their lifetime, and 3% of them will die from it [2]. Breast cancer is a heterogeneous disease; the most common subtype of breast cancer, occurring in about 70% of cancer cases (75% in postmenopausal patients), is hormone-dependent breast cancer [3]. The pharmacological treatment of hormone-dependent early breast cancer in pre-menopausal patients mainly includes the use of tamoxifen for 5–10 years or AIs combined with ovarian suppression. In postmenopausal women, tamoxifen, aromatase inhibitors or their sequences are used, also for a total of 5–10 years. In the case of advanced breast cancer, a combination therapy–aromatase inhibitor or fulvestrant + cyclin-dependent kinase inhibitor CDK4/6, e.g., palbocyclib or tamoxifen/aromatase inhibitor/high dose fulvestrant, is used. In premenopausal women, simultaneous ovarian suppression is necessary [3,4]. Over the past decade, aromatase inhibitors (AIs) became a first-line therapy for patients with hormone-dependent breast cancer because of their greater clinical efficacy and prolonged disease-free survival compared to tamoxifen (TAM) [3]. AIs inhibit the action of the enzyme aromatase. Aromatase (estrogen synthetase) is a member of the cytochrome P450 superfamily of monooxygenases and catalyzes the demethylation of androgens’ carbon 19, producing phenolic 18-carbon estrogens [5]. In postmenopausal women, the major source of estrogen is the conversion of androgens to estrogens in skin, muscle and adipose tissue. Aromatase inhibitors block this pathway, reducing estrogen-mediated cancer cell proliferation in hormone receptor-positive breast cancer [5]. Based on their chemical structure, there are steroidal (exemestane) and non-steroidal (letrozole, anastrozole) aromatase inhibitors [3].

Although AIs have a more favorable risk–benefit profile compared to tamoxifen, such as lower incidence of life-threatening adverse events, for instance thromboembolic episodes and the occurrence of endometrial cancer, they are not free of side effects [5,6]. The most common adverse events during AIs therapy are menopausal symptoms, musculoskeletal events, sexual disorders, impaired cognitive function and bone mineral density (BMD) decline [6]. Moreover, some studies have shown that therapy with aromatase inhibitors is also associated with an increased risk of cardiovascular events and the development of insulin resistance and diabetes [7,8,9]. Patients treated with aromatase inhibitors are more likely to develop hyperlipidemia, hypercholesterolemia and hypertension, which are known risk factors for cardiovascular disease, compared to patients receiving tamoxifen [10]. Some studies showed that treatment with AIs (compared with tamoxifen) was associated with an increased risk of heart failure and cardiovascular mortality [11]. The increase in the incidence of cardiovascular events in patients taking AIs is probably related to the cardioprotective effect of tamoxifen [7]. Nevertheless, the risk of cardiovascular events, dyslipidemia, insulin resistance or diabetes mellitus is increased in postmenopausal women [12]. Epidemiological data show that in the world, 69.1% of all breast cancer cases concern postmenopausal women (data for Western Europe indicate an even higher percentage of 81.4%). At the same time, it is postmenopausal patients who most often die from breast cancer—in the world, as much as 78.8% of all deaths from breast cancer concern postmenopausal patients, while in Western Europe this percentage is as high as 92.9% [13]. Thus, the assessment of whether the risk of developing these disorders additionally increases as a result of therapy with AIs therapy is a significant health concern.

We therefore performed a systematic review and meta-analysis of randomized control trials (RCTs) to determine whether AIs are associated with an increased risk of both cardiovascular and metabolic adverse effects, such as hyperglycemia, dyslipidemia and body weight gain.

## 2. Materials and Methods

This review was conducted in accordance with PRISMA guidelines. Details of the protocol for this systematic review have been registered on PROSPERO and are available at: https://www.crd.york.ac.uk/prospero/display_record.php?ID=CRD42021270743 (accessed on 31 March 2022).

### 2.1. Data Sources and Literature Search

We searched the Cochrane Central Register of Controlled Trials, PubMed (Medline) and EMBASE up to 27 October 2020. The following search terms were used: #1 “breast tumor”, #2 “aromatase inhibitor” OR “anastrozole” “arimidex” OR “letrozole” OR “femara” OR “exemestane” OR “aromasin”, #3 “cardiovascular disease” OR “ischemic heart disease” OR “heart infarction” OR “cerebrovascular accident” OR “body weight” OR “obesity” OR “diabetes mellitus” OR “dyslipidemia” OR “glucose intolerance” OR “insulin resistance” OR “hyperglycemia” OR “hypercholesterolemia” OR “hypertriglyceridemia” OR “metabolic syndrome X”, #4 #1 AND #2 AND #3. No language limitations were applied. Search results in each query were included in supplementary materials (Supplementary Table S1). Reference lists of all included articles were searched to identify potentially relevant articles.

### 2.2. Study Selection and Data Collection Process

Two authors independently (KB and PP) conducted a review of abstracts and titles to remove duplicates and eliminate studies that did not meet the criteria for inclusion in the meta-analysis. Relevant articles were selected by reading the full texts. Disagreements were resolved by discussion among all authors. Inclusion criteria for this meta-analysis were: human, controlled randomized (phase II and III) clinical trials; studies reported on the prevalence of cardiovascular and/or metabolic adverse effects during treatment with third-generation aromatase inhibitor in postmenopausal women with hormone-dependent breast cancer. The comparators could be tamoxifen, placebo or no treatment. The exclusion criteria were: studies reporting on premenopausal women, estrogen or progesterone receptor-negative breast cancer, first/second generation aromatase inhibitors using in the study, reviews, expert opinions, guidelines and case studies. When multiple follow-up periods were reported for a given RCT, we selected the trial with the most comprehensive reporting of cardiovascular or metabolic events and/or the longest follow-up reported. The following data were extracted from included studies with the use of a prespecified data collection form: trial design, trial arm (n included in safety analysis), duration of treatment, characteristics of patients (age, disease stage, primary treatment) and reported adverse effects. In this systemic review, we focused in particular on the frequency of cardiovascular (arterial hypertension, cardiovascular events such as cardiac arrhythmia, ischemic heart disease, myocardial infraction, heart failure, atrial fibrillation) and metabolic adverse effects (hyperglycemia, body weight gain, dyslipidemia). Data were collected by two authors independently (KB and PP) and then compared. Disagreements was resolved by discussion among all authors.

### 2.3. Quality Assessment

The quality assessment was performed with the use of Cochrane Collaboration’s tool [14]. Each RCT was evaluated for selection bias (random sequence generation, allocation concealment), performance bias (blinding of participants), detection bias (blinding of outcome), reporting bias (selective outcome) and other possible bias. Each domain was assigned a “high”, “low” or “unclear” risk of bias independently by two reviewers (KB and PP), with disagreements resolved by discussion among all authors.

### 2.4. Statistical Analysis

**Figure 1:**
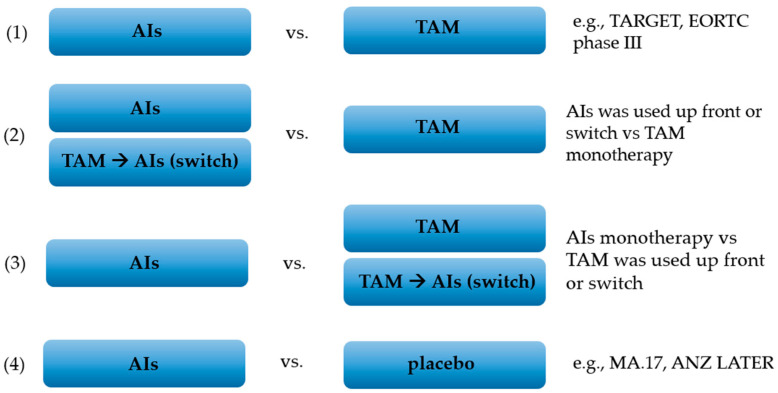
Visualization of groups in analyses. (1) Patients who were only using aromatase inhibitors or tamoxifen for treatment. (2) Group of patients who had been treated with aromatase inhibitors as monotherapy or in sequence with tamoxifen vs. group of patients who had only used tamoxifen for treatment. (3) Patients treated with aromatase inhibitors monotherapy vs. patents treated with tamoxifen alone or in sequence with aromatase inhibitors. (4) Patients treated with aromatase inhibitors vs. those treated with placebo.

A meta-analysis was performed using a random effects model. Odds ratios and 95% confidence intervals (95% CI) were calculated and illustrated using forest plot charts. All analyses were stratified by RCT design. In such a manner, we performed separate analyses depending on the treatment used. In the first analysis, we compared patients treated with aromatase inhibitors only vs. those treated with tamoxifen only (1). In the second analysis, we considered a group of patients who had been treated with aromatase inhibitors as monotherapy or in sequence with tamoxifen and compared them with a group of patients who had only used tamoxifen for treatment (2). The third analysis compared patients treated with aromatase inhibitors monotherapy with those treated with tamoxifen alone or in sequence with aromatase inhibitors (3). The fourth analysis compared patients treated with aromatase inhibitors with those treated with a placebo (4). The principle of selecting studies for each of the analyses is presented in Figure 1.

The following outcomes, cardiovascular events, arterial hypertension, body weight gain and dyslipidemia, were assessed, provided they were reported in at least three studies. Statistical heterogeneity across the RCTs was estimated using the *I*^2^ statistic. All analyses were made using the R-“meta” package version 4.19.0, and all tested *p* values < 0.05 were considered statistically significant.

## 3. Results

**Figure 2:**
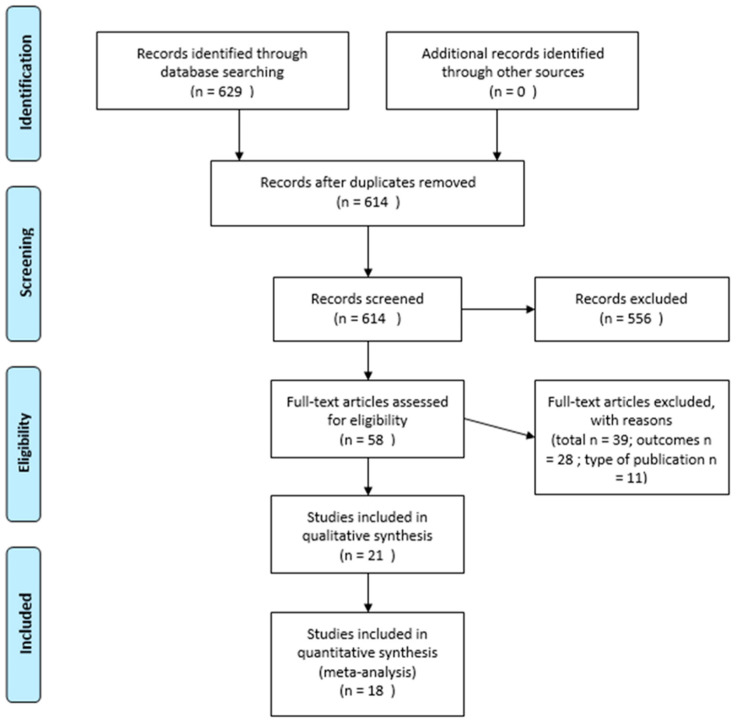
Details of the study selection—PRISMA flow chart of literature search.

Details of the study selection are presented in Figure 2. In total, 614 articles were found. A primary screen of the titles and abstracts resulted in the exclusion of 556 records. A further 39 articles were excluded based on the full-text review. The most common reason for exclusion was inadequate outcomes. This selection produced 21 studies that met the inclusion criteria [15,16,17,18,19,20,21,22,23,24,25,26,27,28,29,30,31,32,33,34,35].

The study design and patient characteristics of included RCTs can be found in Table 1. 

All of the patients were postmenopausal women undergoing treatment for hormone-dependent breast cancer. Most of the research concerned the treatment of early stages of breast cancer (71%). In 13 studies (62%), AIs were used as adjuvant therapy, in 3 studies extended adjuvant therapy, and in the remaining 5 studies first-line treatment for advanced breast cancer. Included studies had a different design; therefore, we decided to analyze the outcomes separately depending on the treatment used: (1) AIs (monotherapy) vs. TAM (monotherapy); (2) AIs (monotherapy) or AIs + TAM (sequence) vs. TAM (monotherapy); (3) AIs (monotherapy) vs. TAM (monotherapy) or AIs + TAM (sequence); and (4) AIs (monotherapy) vs. placebo. Among the studies included, there were three studies that compared the efficacy and safety of two aromatase inhibitors: FACE (letrozole vs. anastrozole), Iwata et al. (exemestane vs. anastrozole) and MA.27 (exemestane vs. anastrozole). However, these studies were not included in the quantitative analysis due to insufficient data to compare the side effect profile for individual aromatase inhibitors.

### 3.1. Quality Assessment

A quality assessment of the included studies using of Cochrane Collaboration’s tool is presented in Supplementary Table S2. The majority of RCTs were of a low risk of bias in different domains of Cochrane Collaboration’s tool. The main limitations regarding the methodological quality were a lack of the blinding of participants and outcome.

### 3.2. Cardiovascular Events

**Figure 3:**
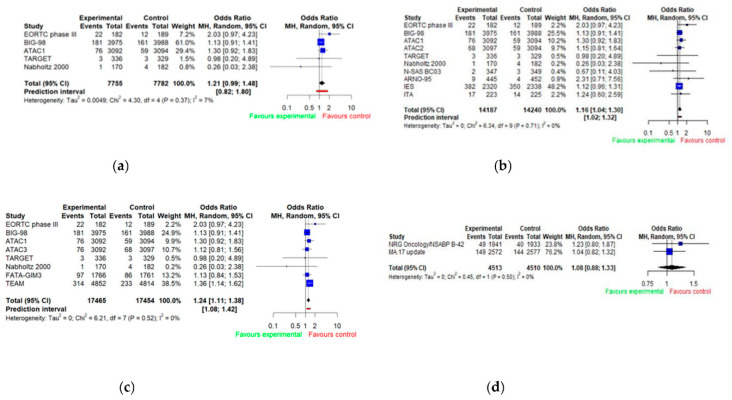
Forest plot of odds ratios for cardiovascular events with AIs by trial design (**a**–**d**). E (experimental group); C (control group). (**a**) E: AIs vs. C: tamoxifen (monotherapy); (**b**) E: AIs (monotherapy) or AIs + tamoxifen (sequence) vs. C: tamoxifen (monotherapy); (**c**) E: AIs (monotherapy) vs. C: AIs + tamoxifen (sequence) or tamoxifen (monotherapy); (**d**) E: AIs (monotherapy) vs. C: placebo/no treatment.

Cardiovascular events included all reported events of ischemic heart disease, myocardial infarction, heart failure, atrial fibrillation or cardiac arrhythmia. Pooled analysis-of-odds ratios for cardiovascular events are presented in Figure 3 (Figure 3a–d). Thirteen studies reported adverse effects classified as cardiovascular events. A pooled analysis of five trials comparing AIs to tamoxifen showed the odds for cardiovascular events being 1.21 times higher for patients taking aromatase inhibitor, however not statistically significantly (OR = 1.21; 95% CI 0.99–1.48). The heterogeneity across studies was low, with *I*^2^ = 7% (*p* = 0.37) (Figure 3a). In the ATAC trial, there were three arms–patients receiving aromatase inhibitor (anastrozole), patients receiving tamoxifen, or patients taking both anastrozole and tamoxifen. ATAC1 relates to the situation when aromatase inhibitor alone was compared with tamoxifen alone, ATAC2 relates to the situation when aromatase inhibitor used in combination with tamoxifen was compared with tamoxifen alone, and ATAC3 relates to the situation when aromatase inhibitor alone was compared with the combination of aromatase inhibitor and tamoxifen.

Comparing study arms in which aromatase inhibitors were used (alone or in sequence with tamoxifen) with the arms in which tamoxifen was used alone (*n* = 9) allowed for finding a statistically higher risk of cardiovascular events for AIs (OR = 1.16; 95% CI 1.04–1.30). The heterogeneity across studies was low: *I*^2^ = 0% (*p* = 0.71) (Figure 3b). Similar results were observed when comparing patients taking aromatase inhibitors alone to patients taking tamoxifen alone or in sequence with aromatase inhibitor (OR = 1.24; 95% CI 1.11–1.38) (Figure 3c). Two studies that compared patients taking aromatase inhibitor as an extended adjuvant therapy with those on placebo showed no difference in relation to the occurrence of cardiovascular events (OR = 1.08; 95% CI 0.88–1.33) (Figure 3d).

### 3.3. Arterial Hypertension

**Figure 4:**
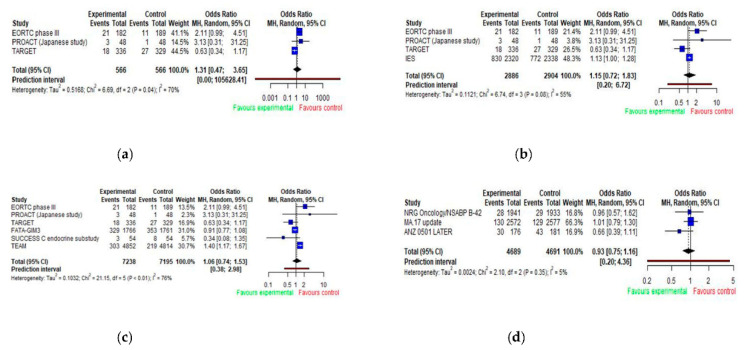
Forest plot of odds ratios for arterial hypertension with AIs by trial design (**a**–**d**). E (experimental group); C (control group). (**a**) E: AIs vs. C: tamoxifen (monotherapy); (**b**) E: AIs (monotherapy) or AIs + tamoxifen (sequence) vs. C: tamoxifen (monotherapy); (**c**) E: AIs (monotherapy) vs. C: AIs + tamoxifen (sequence) or tamoxifen (monotherapy); (**d**) E: AIs (monotherapy) vs. C: placebo/no treatment.

Ten studies reported arterial hypertension as an adverse effect. Pooled analyses of odds ratios for arterial hypertension are presented in Figure 4 (Figure 4a–d). An analysis of five trials comparing adjuvant AIs to tamoxifen showed the odds for arterial hypertension being 1.31 times higher for patients taking aromatase inhibitor; however, this did not reach statistical significance (OR = 1.31; 95% CI 0.47–3.65). The studies showed high heterogeneity, with *I*^2^ = 70% (*p* = 0.04) (Figure 4a).

There was no difference when comparing patients taking aromatase inhibitors alone or sequentially with tamoxifen to patients taking tamoxifen alone (OR = 1.15; 95% CI 0.72–1.83) (Figure 4b); patients taking aromatase inhibitors alone to patients taking tamoxifen alone or sequential with aromatase inhibitor (OR = 1.06; 95% CI 0.74–1.53) (Figure 4c); and, similarly, patients on aromatase inhibitor as extended adjuvant therapy to those on placebo (OR = 0.93; 95% CI 0.75–1.16) (Figure 4d). The highest OR for arterial hypertension of 1.40 (95% CI 1.17–1.67) was reported in the TEAM study.

### 3.4. Body Weight Gain

**Figure 5:**
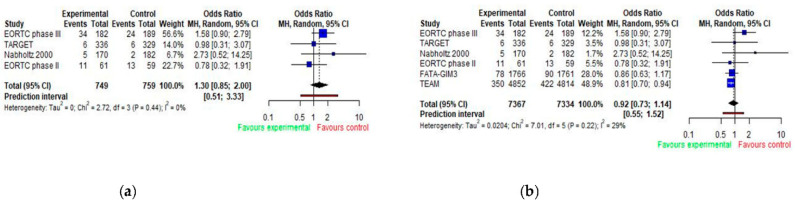
Forest plot of odds ratios for body weight gain with AIs by trial design (**a**,**b**). E (experimental group); C (control group). (**a**) E: AIs vs. C: tamoxifen (in monotherapy); (**b**) E: AIs (monotherapy) vs. C: AIs + tamoxifen (sequence) or tamoxifen (monotherapy).

Body weight gain as an adverse effect was reported in six studies. Pooled analyses of odds ratios for body weight gain are presented in Figure 5 (Figure 5a,b). The results were not statistically significant: OR = 1.30; 95% CI 0.85–3.33 when compared patients taking aromatase inhibitor to patients taking tamoxifen, and OR = 0.92; 95% CI 0.73–1.14 when compared patients taking aromatase inhibitor to patients taking tamoxifen alone or sequentially with aromatase inhibitor (Figure 5a,b).

### 3.5. Dyslipidemia

**Figure 6:**
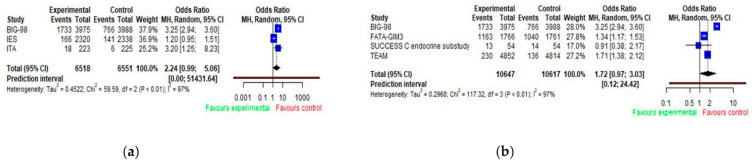
Forest plot of odds ratios for dyslipidemia with AIs by trial design (**a**,**b**). E (experimental group); C (control group). (**a**) E: AIs (monotherapy) or AIs + tamoxifen (sequence) vs. C: tamoxifen (monotherapy); (**b**) E: AIs (monotherapy) vs. C: AIs + tamoxifen (sequence) or tamoxifen (monotherapy).

In our study we defined dyslipidemia as all lipid disorders, such as hyperlipidemia, hypercholesterolemia or hypertriglyceridemia. Dyslipidemia as an adverse effect was reported in six studies. Pooled analyses of odds ratios for dyslipidemia are presented in Figure 6 (Figure 6a,b). When data were pooled across trials, no evidence of difference was observed. However, these analyses were inconclusive due to wide 95% CIs (aromatase inhibitor alone or sequentially with tamoxifen vs. tamoxifen: OR = 2.24; 95% CI 0.99–5.06; aromatase inhibitor alone vs. tamoxifen alone or sequentially with aromatase inhibitor: OR = 1.72; 95% CI 0.97–3.03) and high heterogeneity across studies with I2 = 97% (Figure 6a,b). The highest OR for dyslipidemia of 3.25 (95% CI 2.94–3.60) was reported in the BIG-98 study.

## 4. Discussion

The aim of this study was to determine whether treatment using AIs is associated with an increased risk of both cardiovascular and metabolic adverse effects, such as body weight gain, dyslipidemia, hyperglycemia or insulin resistance. Due to the fact that treatment with AIs is mainly used in postmenopausal women and given that these patients, due to their post-menopausal status, are more likely to suffer from cardiovascular events, lipid metabolism disorders or diabetes mellitus [12], this is a significant health concern.

To our knowledge, this is the most recent study to assess the cardiovascular and metabolic risk of AIs treatment in breast cancer patients. We found that treatment with AIs (alone or in sequence with tamoxifen) increases the risk for cardiovascular events (OR = 1.16; 95% CI 1.04–1.30) comparing with tamoxifen alone. Similar results were observed when patients taking aromatase inhibitors alone were compared to those taking tamoxifen alone or in sequence with aromatase inhibitor (OR = 1.24; 95% CI 1.11–1.38). Our findings are consistent with the results of the study by Khosrow-Khavar et al., who showed that the use of AIs compared to tamoxifen was associated with a 19% increase in the risk of cardiovascular adverse events (RR = 1.19, 95% CI 1.07–1.34) [7]. In contrast, we separately analyzed the studies depending on the treatment regimens of tamoxifen alone vs. AI alone (no relationship; OR = 1.21; 95% CI 0.99–1.48), AI alone vs. tamoxifen alone or sequentially with AI (e.g., the TEAM study), and AI alone or sequentially with tamoxifen vs. tamoxifen alone (e.g., the IES study). The lack of statistical significance in the comparison of tamoxifen alone vs. aromatase inhibitor alone could be explained by the small number of studies with this kind of design. Khosrow-Khavar et al. hypothesized that the increase in the incidence of cardiovascular events in patients treated with AIs is probably related to the cardioprotective effect of tamoxifen, which seems also be the case in our findings as there was no difference when the aromatase inhibitor treatment group was compared to the placebo treatment group.

The occurrence of cardiovascular events may also depend on the duration of therapy and the type of AI. Results from a population-based cohort study conducted by Sund et al. indicated an increased risk for arrhythmia and acute ischemic heart disease in patients treated for more than four years with AIs [36]. In turn, a network meta-analysis performed by Zhao et al. showed the total and severe cardiovascular events’ risk ranking for letrozole, exemestane and anastrozole in descending order [37]. Different results were presented by He et al. [38], who suggested that patients treated with AIs do not have a significant risk of developing cardiovascular events in comparison with tamoxifen treatment (OR = 0.9940, 95% CI 0.8545–1.1562). However, in the same study it was found that almost all of the high-grade cardiovascular events occurred in patients treated with AIs [38].

Regardless of the treatment regimen, we did not show a significantly increased risk for arterial hypertension to be associated with AIs. Nevertheless, such a risk was reported in the TEAM study with an OR of 1.40 (95% CI 1.17–1.67) [22]. Blaes et al., examining vascular function in breast cancer patients, showed that compared to healthy post-menopausal women, women on AI had a higher mean systolic blood pressure (128.6 mmHg vs. 116.2 mmHg; *p* = 0.004) [39].

As with arterial hypertension, we did not show an increased risk for body weight gain in the AI-treated groups. Sestak et al. analyzed, for weight change, three large clinical trials (ATAC, IBIS-I, IBIS-II) and they reported that weight gain did not differ between AIs, tamoxifen and placebo [40], which is consistent with the results of the current meta-analysis. The body weight was compared at 2 years with that at diagnosis in 625 patients with breast cancer, and 31% had lost > 2 kg, 34% had a stable weight and 35% had gained >2 kg. Main factors associated with > 2 kg weight gain were pre-menopausal status and receiving any chemotherapy [41].

Patients treated with aromatase inhibitor tended to have a higher risk for dyslipidemia than those treated with tamoxifen, though the difference did not reach statistical significance. It could have been due to wide 95% CIs and high heterogeneity across studies (*I*^2^ = 97%). The highest risk was reported in the BIG-98 study (OR = 3.25; 95% CI 2.94–3.60). A total of 43.6% of patients in the letrozole group and 19.2% of patients in the tamoxifen group had hypercholesterolemia recorded at least once during treatment [28]. Wang et al. conducted a prospective single-center cohort study and found that steroidal aromatase inhibitor (exemestane) had a more favorable effect on lipid profiles than nonsteroidal aromatase inhibitors (anastrozole, letrozole). The cumulative incidence of lipid events in the steroidal and nonsteroidal groups at 24 months was 25.3% and 37.0%, respectively [42]. These findings are consistent with the results obtained in the BIG-98 study [28].

Data to assess the odds for AI-associated hyperglycemia was insufficient to perform a meta-analysis. Hyperglycemia was reported as an adverse event only in five studies having a different drug regimen. In the ITA study, hyperglycemia was observed in 1.3% of patients receiving tamoxifen therapy for 5 years and 4.5% of patients receiving tamoxifen and anastrozole sequentially [27]. In contrast, in the SUCCESS C study, hyperglycemia was significantly more frequent in patients receiving sequential therapy with tamoxifen and AI than in patients receiving therapy with exemestane alone (28% vs. 22%) [16]. In the study by Iwata et al., comparing exemestane and anastrozole in the first-line treatment of advanced breast cancer, the proportion of patients with hyperglycemia was significantly higher in the exemestane group, at 51.4% and 47.7%, respectively [18]. Despite the lack of data from large clinical trials on carbohydrate disturbances in patients using aromatase inhibitors, this seems to be an important issue. Gibb et al., in a case-control study, compared women with breast cancer diagnoses and receiving aromatase inhibitor therapy with age-matched healthy control subjects. They found that aromatase inhibitor therapy was associated with significantly lower insulin sensitivity, higher peak insulin concentration after oral glucose tolerance test, greater percentage of body fat and higher plasma leptin concentration [8]. In turn, Hamood et al. investigated the association between hormone therapy and diabetes risk in breast cancer survivors. Of 2246 breast cancer survivors, 324 developed diabetes over a mean follow-up of 5.9 years. They found the hazard for aromatase inhibitor use (HR = 4.27; 95% CI 1.42–12.84; *p* = 0.010) being higher than for the use of tamoxifen (HR = 2.25; 95% CI 1.19–4.26; *p* = 0.013) [9]. Different results come from the meta-analysis by Feng et al. exploring the association of hormone therapy (HT) and secondary diabetes in breast cancer patients. They showed that HT significantly increased the risk of developing diabetes mellitus. However, when analyzing specific HT medications, TAM use significantly enhanced the incidence of secondary diabetes mellitus, while AIs use did not have an influence [43].

Our study is not without limitations. First, the meta-analysis was based on the results of primary studies, not on individual patient data. We included studies with postmenopausal women with breast cancer of any stage–early, metastatic or advanced. Patients with metastatic or advanced BC may have been exposed to a greater number of prior treatments, e.g., chemotherapy, which could also cause cardiotoxicity. To minimize the impact of this factor, we performed an additional analysis of early vs. advanced / metastatic breast cancer. The results of this analysis can be found in Supplementary Material S1. Then, there was a heterogeneity in reporting adverse effects between studies. The results of the sensitivity analysis can be found in Supplementary Material S2. There was also a heterogeneity across RCTs concerning the duration of follow-up and the trial design. However, to minimize the impact of the treatment regimen, we decided to conduct separate analyses in this respect. In addition, we did not have enough information characterizing patients at baseline in terms of the presence of cardiovascular or metabolic disorders. All of this may jeopardize our results to a certain extent.

## 5. Conclusions

In conclusion, our results suggest that postmenopausal women with breast cancer treated with AIs have an increased risk of cardiovascular events in comparison to those treated with tamoxifen, which is largely due to the cardioprotective effect of the latter compared to the cardiotoxicity of AIs. We were unable to find a similar association for hypertension, dyslipidemia, hyperglycemia, insulin resistance or weight gain. Further large, high-quality RCTs and post-marketing safety observational studies are still needed to definitively evaluate the impact of AIs on cardiovascular events and metabolic disorders in breast cancer patients.

## Figures and Tables

**Table 1:** Description of included studies. Legend: TAM—tamoxifen, EXE—exemestane, LET—letrozole, ANA—anastrozole, PBO—placebo, comb—combination, observ—observation, ND—no data.

TRIAL	TRIAL ARM (*n* Included in Safety Analysis)	TREATMENT	TRIAL DESIGN	AGE (Mean)	CANCER STAGE	PRIMARY TREATMENT		
						Surgery (%)	Radiotherapy (%)	Systemic Therapy (%)
**FATA-GIM3** [15]	switch group = 1761 upfront group = 1766	adjuvant	upfront strategy vs. switch strategy; six treatment groups: ANA 1 mg, EXE 25 mg, LET 2,5 mg for 5 years; TAM 20 mg for 2 years followed by administration ANA or EXE or LET for 3 years	64	early	100%	1247 (67%)	712 (39%)
	upfront group = 1766					100%	1253 (68%)	703 (38%)
	ANA = 1175					100%	801 (65%)	469 (39%)
	EXE = 1177					100%	854 (69%)	474 (38%)
	LET = 1175					100%	845 (69%)	472 (39%)
**SUCCESS C** [16]	EXE = 54	adjuvant	5 years EXE vs. 2 years TAM + 3 years EXE	EXE-63	early	ND	ND	100%
	TAM-EXE = 54			TAM-EXE -60.5		ND	ND	100%
**FACE** [17]	LET = 2049	adjuvant	LET (2.5 mg) vs. ANA (1 mg) for 5 years	62	early	ND	652 (31.6%)	1294 (62.7%)
	ANA = 2062					ND	621 (29.9%)	1267 (61.1%)
**Iwata et al, 2013** [18]	EXE = 149	first-line	EXE 25 mg vs. ANA 1 mg continued until disease progression, intolerable adverse event or death	EXE-63.4	advanced	ND	35 (23.5%)	103 (69.1%)
	ANA = 149			ANA-64		ND	28 (18.8%)	100 (67.1%)
**MA.27** [19]	EXE = 3761	adjuvant	EXE 25 mg vs. ANA 1 mg for 5 years	EXE-63.9	early	3789 (100%)	ND	1163 (31%)
	ANA = 3759			ANA-64.3		3787 (100%)	ND	1164 (31%)
**PROACT** [20]	ANA = 48	neoadjuvant and adjuvant	pre-operative (3 months) and post-operative (5 years or until recurrence, withdrawal) treatment TAM (20 mg) vs. ANA (1 mg)	ANA-61.5	locally advanced	48 (100%)	18 (41.9%)	10 (23.3%)
	TAM = 48			TAM-61.6		49 (100%)	17 (39.5%)	20 (46.5%)
**N-SAS BC04** [21]	EXE = 55	adjuvant	EXE for 5 years vs. 2.5–3 years TAM followed by EXE to a total of 5 years vs. ANA for 5 years	EXE-63.2	early	15 (27.3%)	35 (63.6%)	21 (38.2%)
	TAM = 56			TAM-63.0		18 (32.1%)	36 (64.3%)	23 (41.1%)
	ANA = 55			ANA-62.9		18 (32.7%)	34 (61.8%)	21 (38.2%)
**TEAM** [22]	TAM = 4814	adjuvant	25 mg EXE vs. TAM (20 mg) --> EXE for 5 years (EXE after 2.5–3 years TAM)	TAM ≥ 50–97%	early	4868 (100%)	3320 (68%)	1740 (36%)
	EXE = 4852			EXE ≥ 50–97%		4898 (100%)	3377 (69%)	1773 (36%)
**N-SAS BC03** [23]	TAM = 349	adjuvant	TAM for 5 years vs. TAM (20 mg) for 1–4 years --> ANA (1 mg) to complete 5 years of hormone therapy	TAM-60.2	early	349 (100%)	ND	186 (53.3%)
	ANA = 347			ANA-59.5		347 (100%)	ND	187 (53.9%)
**EORTC phase III, 2008** [24]	EXE = 182	first-line	TAM 20 mg vs. EXE 25 mg until disease progression or unacceptable toxicity occurred	EXE-63	metastatic	ND	75 (41.2%)	76 (41.7%)
	TAM = 189			TAM-62		ND	79 (41.8%)	79 (41.8%)
**ARNO-95** [25]	ANA = 445	adjuvant	TAM for 5 years vs. TAM for 2 years --> ANA for 3 years	ANA-60.9	early	489 (100%)	326 (66.7%)	ND
	TAM = 452			TAM-60.5		490 (100%)	332 (67.8%)	ND
**IES** [26]	EXE = 2320	adjuvant	TAM 20 mg for 5 years vs. TAM 20 mg for 2 or 3 years, then switch to EXE 25 mg to complete a total of five years of adjuvant endocrine treatment	EXE-64.3	early	2349 (99.9%)	ND	766 (32.4%) chemoth.; 567 (24.0%) hormone-th.
	TAM = 2338			TAM-64.2		2365 (99.7%)	ND	765 (32.1%) chemoth.; 557 (23.4%) hormone-th.
**ITA** [27]	TAM = 225	adjuvant	TAM 20 mg (2–3 years) --> ANA 1 mg to complete 5-years treatment vs. TAM 20 mg for 5 years	63	early	225 (100%)	110 (49%)	150 (67%)
	ANA = 223					223 (100%)	120 (54%)	149 (67%)
**BIG-98** [28]	LET (LET for 5 years; LET --> TAM) = 3975	adjuvant	LET (2.5 mg) vs. TAM (20 mg) vs. LET (2 years) --> TAM (3 years) vs. TAM (2 years) --> LET (3 years) for 5 years (this analysis compares the two groups assigned to receive LET initially with the two groups assigned to receive TAM initially)	61	early	4003 (100%)	2867 (71.6%)	1012 (25.3%)
	TAM (TAM for 5 years; TAM --> LET) = 3988					4007 (100%)	2870 (71.6%)	1012 (25.3%)
**MA.17** [29]	LET = 2572	extended adjuvant	LET (2.5 mg) vs. placebo for 5 years	LET-62.0	early	1286 (50%)	1550 (60%)	1175 (46%)
	PBO = 2577			PBO-62.0		1301 (50%)	1528 (59%)	1177 (46%)
**EORTC phase II trial** [30]	EXE = 61	first-line	TAM 20 mg vs. EXE 25 mg; treatment was continued until progression of disease, unacceptable toxity, patient refusal or start of any new anti-cancer therapy	EXE-62	metastatic	ND	59%	42%
	TAM = 59			TAM-63		ND	59%	43%
**ATAC** [31]	ANA = 3092	adjuvant	ANA 1 mg + TAM placebo vs. ANA placebo + TAM 20 mg vs. ANA 1 mg + TAM 20 mg for 5 years	ANA-64.1	early	1494 (47.8%)	1978 (63.3%)	698 (22.3%)
	TAM = 3094			TAM-64.1		1474 (47.3%)	1946 (62.5%)	647 (20.8%)
	comb = 3097			comb-64.3		1502 (48.1%)	1936 (62.0%)	651 (20.8%)
**TARGET** [32]	ANA = 336	first-line	ANA 1 mg vs. TAM 20 mg; trial treatment was continued until disease progression	ANA-67	advanced	ND	ND	105 (30.8%)
	TAM = 329			TAM-66		ND	ND	97 (29.6%)
**Nabholtz et al., 2000** [33]	ANA = 170	first-line	ANA 1 mg vs. TAM 20 mg; trial treatment was continued until disease progression	ANA-68	advanced	ND	ND	68 (39.8%)
	TAM = 182			TAM-67		ND	ND	70 (38.4%)
**NRG Oncology/NSABP B-42** [34]	PBO = 1933	extended adjuvant	LET 2.5 mg vs. placebo for 5 years	ND	early	775 (39.1%)	ND	ND
	LET = 1941			ND		782 (39.4%)	ND	ND
**ANZ 0501 LATER** [35]	observ = 181	extended adjuvant	LET 2.5 mg for 5 years vs. observation	observ-64	early	67 (37.4%)	126 (70.4%)	86 (48.0%)
	LET = 176			LET- 65		64 (35.4%)	130 (71.8%)	75 (41.4%)

## Data Availability

All data are available in the article and supplementary material. We will willingly share our knowledge, protocol and expertise when asked.

## Supplementary Materials

The following supporting information can be downloaded at: https://www.mdpi.com/article/10.3390/jcm11113133/s1, Supplementary Table S1: Search results; Supplementary Table S2: Quality assessment of included studies. Supplementary Material S1: sensitivity analysis. Supplementary Material S2: additional analysis early vs. advanced/metastatic breast cancer.
