# Supplementary material for: Aromatase Inhibitors and Risk of Metabolic and Cardiovascular Adverse Effects in Breast Cancer Patients—A Systematic Review and Meta-Analysis
Source: J Clin Med. 2022 May 31;11(11):3133. doi: 10.3390/jcm11113133 (PMC9181297; doi:10.3390/jcm11113133)
Supplement: Supplementary file 1 [file jcm-11-03133-s001.zip › Supplementary Material S1.pdf]

Supplementary Material S1: additional analysis early vs. advanced/metastatic breast cancer.

An additional analysis was performed only when the qualified studies included studies involving patients with early and advanced breast cancer.

1. **Cardiovascular events:**

A. AIs vs. tamoxifen monotherapy.

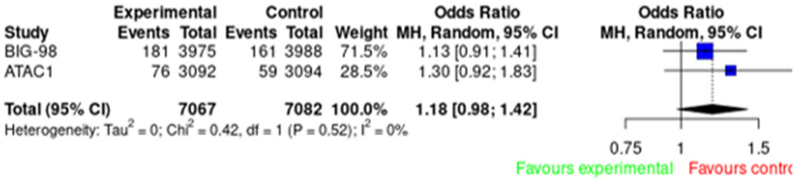

EARLY BREAST CANCER

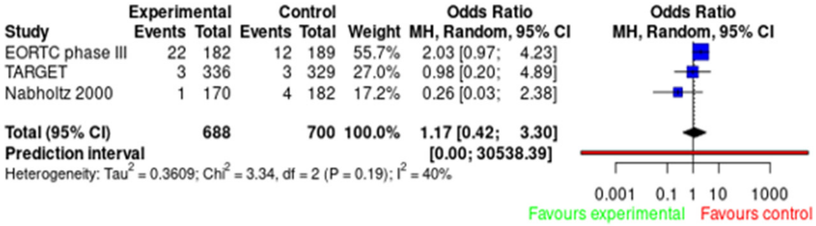

ADVANCED/METASTATIC BREAST CANCER

B. AIs (monotherapy) or AIs+tamoxifen (sequence) vs. tamoxifen (monotherapy).

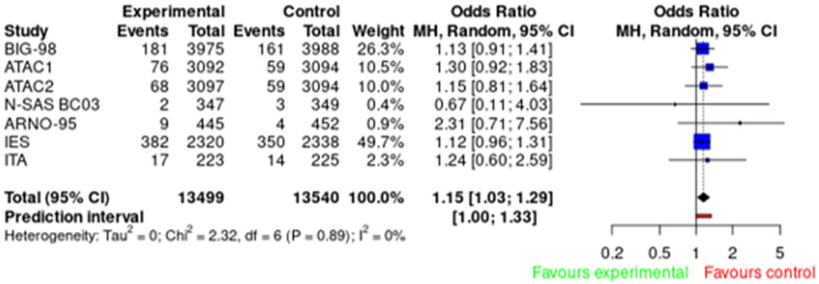

EARLY BREAST CANCER

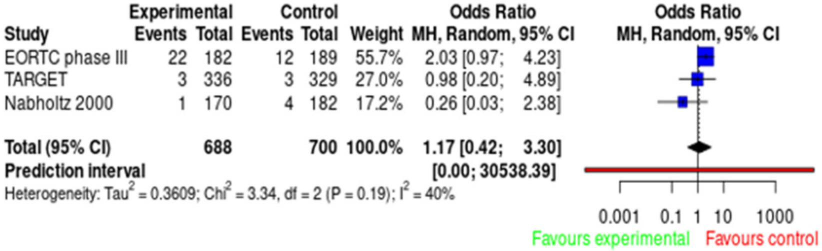

ADVANCED/METASTATIC BREAST CANCER

C. AIs (monotherapy) vs. AIs+tamoxifen (sequence) or tamoxifen (monotherapy).

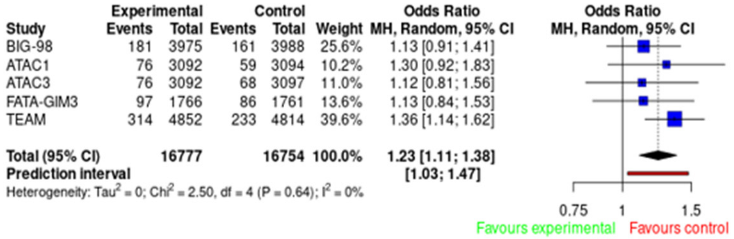

EARLY BREAST CANCER

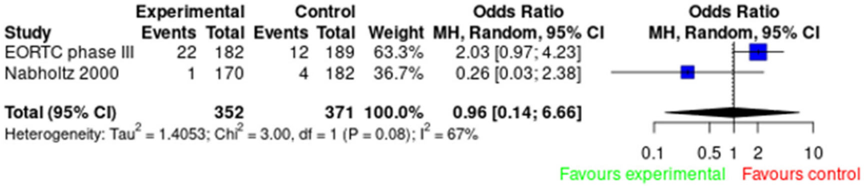

ADVANCED/METASTATIC BREAST CANCER

2. Arterial hypertension:

A. AIs vs. tamoxifen monotherapy.

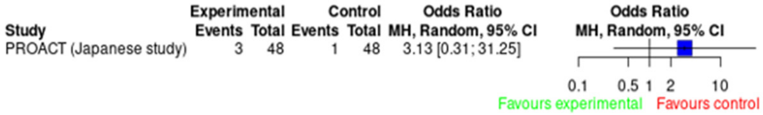

EARLY BREAST CANCER

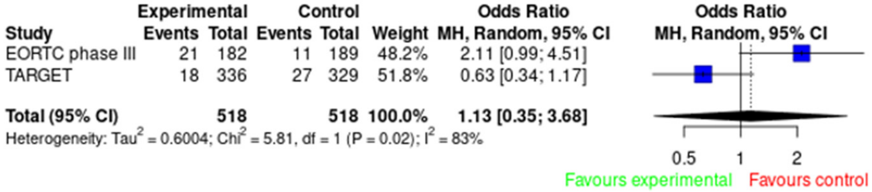

ADVANCED/METASTATIC BREAST CANCER

B. AIs (monotherapy) or AIs+tamoxifen (sequence) vs. tamoxifen (monotherapy).

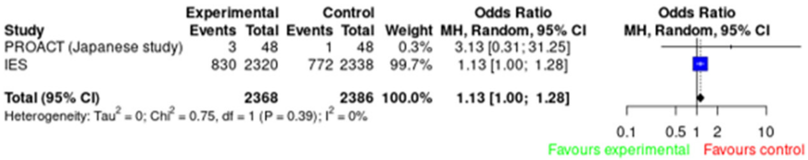

EARLY BREAST CANCER

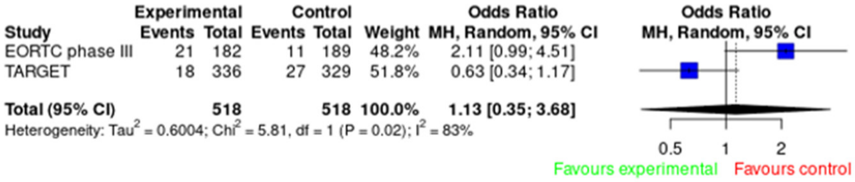

ADVANCED/METASTATIC BREAST CANCER

C. AIs (monotherapy) vs. AIs+tamoxifen (sequence) or tamoxifen (monotherapy).

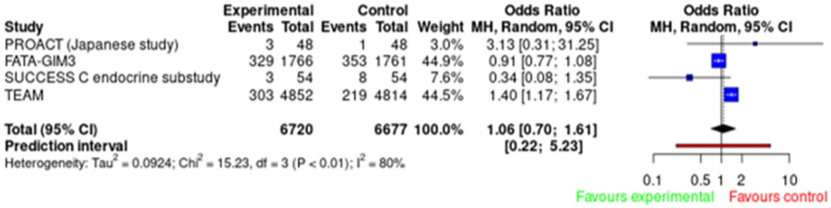

EARLY BREAST CANCER

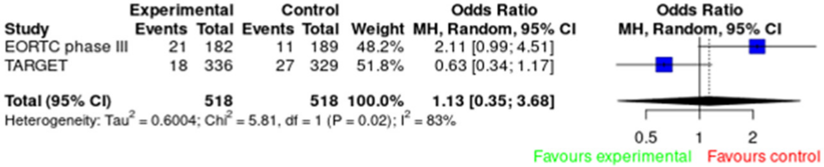

ADVANCED/METASTATIC BREAST CANCER

3. Body weight gain:

A. AIs (monotherapy) vs. AIs+tamoxifen (sequence) or tamoxifen (monotherapy)

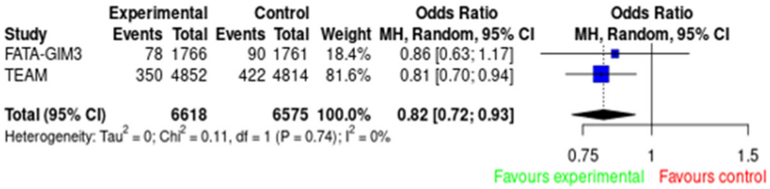

EARLY BREAST CANCER

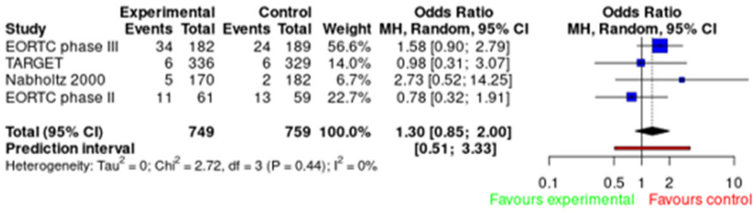

ADVANCED/METASTATIC BREAST CANCER
