# Supplementary material for: Aromatase Inhibitors and Risk of Metabolic and Cardiovascular Adverse Effects in Breast Cancer Patients—A Systematic Review and Meta-Analysis
Source: J Clin Med. 2022 May 31;11(11):3133. doi: 10.3390/jcm11113133 (PMC9181297; doi:10.3390/jcm11113133)

Supplementary Material S2: sensitivity analysis.

In the sensitivity analysis, were made the assumptions that studies with incidence rate of events < 0,01 (1%) will be exluded, therefore it only applies to cardiovascular events.

Cardiovascular events:

1. AIs vs. tamoxifen (monotherapy) – all (left) vs. without low incidence (right)

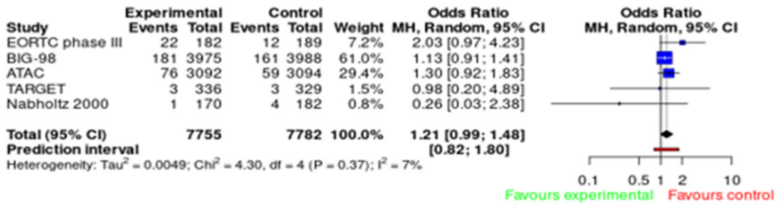

Study	Experimental Events	Experimental Total	Control Events	Control Total	Weight	Odds Ratio MH, Random, 95% CI
EORTC phase III	22	182	12	189	7.2%	2.03 [0.97; 4.23]
BIG-98	181	3975	161	3988	61.0%	1.13 [0.91; 1.41]
ATAC	76	3092	59	3094	29.4%	1.30 [0.92; 1.83]
TARGET	3	336	3	329	1.5%	0.98 [0.20; 4.89]
Nabholtz 2000	1	170	4	182	0.8%	0.26 [0.03; 2.38]
**Total (95% CI)**		**7755**		**7782**	**100.0%**	**1.21 [0.99; 1.48]**
**Prediction interval**						**[0.82; 1.80]**

Heterogeneity: $Tau^2 = 0.0049$; $Chi^2 = 4.30$, df = 4 (P = 0.37); $I^2 = 7\%$

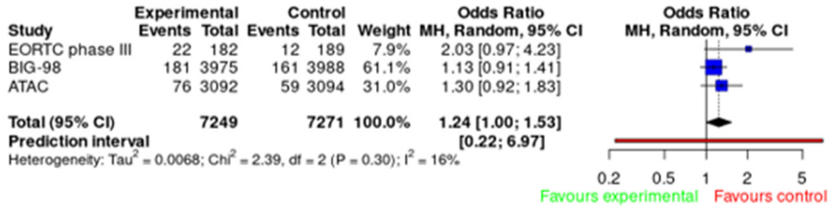

Study	Experimental Events	Experimental Total	Control Events	Control Total	Weight	Odds Ratio MH, Random, 95% CI
EORTC phase III	22	182	12	189	7.9%	2.03 [0.97; 4.23]
BIG-98	181	3975	161	3988	61.1%	1.13 [0.91; 1.41]
ATAC	76	3092	59	3094	31.0%	1.30 [0.92; 1.83]
**Total (95% CI)**		**7249**		**7271**	**100.0%**	**1.24 [1.00; 1.53]**
**Prediction interval**						**[0.22; 6.97]**

Heterogeneity: $Tau^2 = 0.0068$; $Chi^2 = 2.39$, df = 2 (P = 0.30); $I^2 = 16\%$

2. AIs (monotherapy) or AIs+tamoxifen (sequence) vs. tamoxifen (monotherapy) - all (left) vs. without low incidence (right)

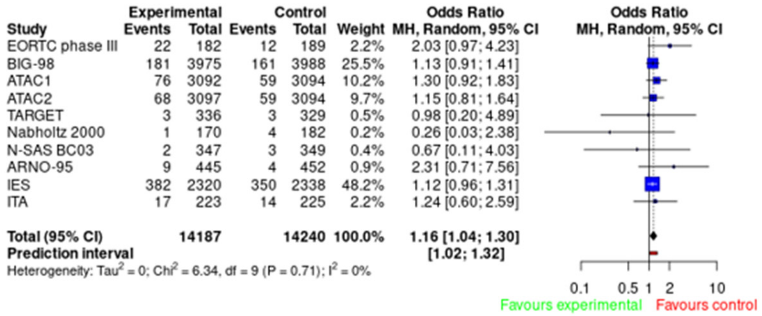

Study	Experimental Events	Experimental Total	Control Events	Control Total	Weight	Odds Ratio MH, Random, 95% CI
EORTC phase III	22	182	12	189	2.2%	2.03 [0.97; 4.23]
BIG-98	181	3975	161	3988	25.5%	1.13 [0.91; 1.41]
ATAC1	76	3092	59	3094	10.2%	1.30 [0.92; 1.83]
ATAC2	68	3097	59	3094	9.7%	1.15 [0.81; 1.64]
TARGET	3	336	3	329	0.5%	0.98 [0.20; 4.89]
Nabholtz 2000	1	170	4	182	0.2%	0.26 [0.03; 2.38]
N-SAS BC03	2	347	3	349	0.4%	0.67 [0.11; 4.03]
ARNO-95	9	445	4	452	0.9%	2.31 [0.71; 7.56]
IES	382	2320	350	2338	48.2%	1.12 [0.96; 1.31]
ITA	17	223	14	225	2.2%	1.24 [0.60; 2.59]
**Total (95% CI)**		**14187**		**14240**	**100.0%**	**1.16 [1.04; 1.30]**
**Prediction interval**						**[1.02; 1.32]**

Heterogeneity: $Tau^2 = 0$; $Chi^2 = 6.34$, df = 9 (P = 0.71); $I^2 = 0\%$

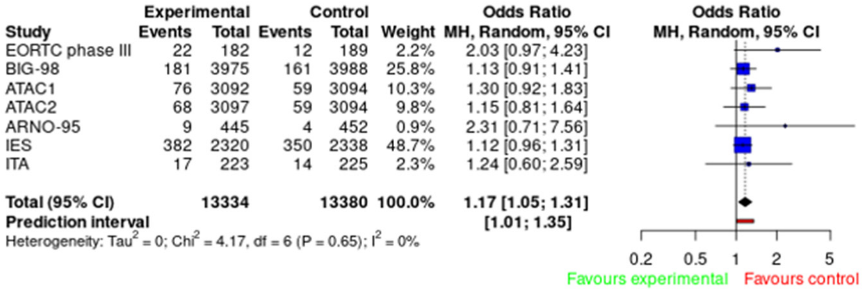

Study	Experimental Events	Experimental Total	Control Events	Control Total	Weight	Odds Ratio MH, Random, 95% CI
EORTC phase III	22	182	12	189	2.2%	2.03 [0.97; 4.23]
BIG-98	181	3975	161	3988	25.8%	1.13 [0.91; 1.41]
ATAC1	76	3092	59	3094	10.3%	1.30 [0.92; 1.83]
ATAC2	68	3097	59	3094	9.8%	1.15 [0.81; 1.64]
ARNO-95	9	445	4	452	0.9%	2.31 [0.71; 7.56]
IES	382	2320	350	2338	48.7%	1.12 [0.96; 1.31]
ITA	17	223	14	225	2.3%	1.24 [0.60; 2.59]
**Total (95% CI)**		**13334**		**13380**	**100.0%**	**1.17 [1.05; 1.31]**
**Prediction interval**						**[1.01; 1.35]**

Heterogeneity: $Tau^2 = 0$; $Chi^2 = 4.17$, df = 6 (P = 0.65); $I^2 = 0\%$

3. AIs (monotherapy) vs. AIs+tamoxifen (sequence) or tamoxifen (monotherapy) - all (left) vs. without low incidence (right)

Study	Experimental Events	Experimental Total	Control Events	Control Total	Weight	Odds Ratio MH, Random, 95% CI
EORTC phase III	22	182	12	189	2.2%	2.03 [0.97; 4.23]
BIG-98	181	3975	161	3988	24.9%	1.13 [0.91; 1.41]
ATAC1	76	3092	59	3094	9.9%	1.30 [0.92; 1.83]
ATAC2	76	3092	68	3097	10.7%	1.12 [0.81; 1.56]
TARGET	3	336	3	329	0.5%	0.98 [0.20; 4.89]
Nabholtz 2000	1	170	4	182	0.2%	0.26 [0.03; 2.38]
FATA-GIM3	97	1766	86	1761	13.2%	1.13 [0.84; 1.53]
TEAM	314	4852	233	4814	38.5%	1.36 [1.14; 1.62]
**Total (95% CI)**		**17465**		**17454**	**100.0%**	**1.24 [1.11; 1.38]**
**Prediction interval**						**[1.08; 1.42]**

Heterogeneity: $Tau^2 = 0$; $Chi^2 = 6.21$, df = 7 (P = 0.52); $I^2 = 0\%$

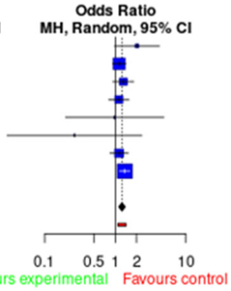

Study	Experimental Events	Experimental Total	Control Events	Control Total	Weight	Odds Ratio MH, Random, 95% CI
EORTC phase III	22	182	12	189	2.2%	2.03 [0.97; 4.23]
BIG-98	181	3975	161	3988	25.0%	1.13 [0.91; 1.41]
ATAC1	76	3092	59	3094	10.0%	1.30 [0.92; 1.83]
ATAC2	76	3092	68	3097	10.8%	1.12 [0.81; 1.56]
FATA-GIM3	97	1766	86	1761	13.3%	1.13 [0.84; 1.53]
TEAM	314	4852	233	4814	38.8%	1.36 [1.14; 1.62]
**Total (95% CI)**		**16959**		**16943**	**100.0%**	**1.25 [1.12; 1.39]**
**Prediction interval**						**[1.07; 1.45]**

Heterogeneity: $Tau^2 = 0$; $Chi^2 = 4.22$, df = 5 (P = 0.52); $I^2 = 0\%$

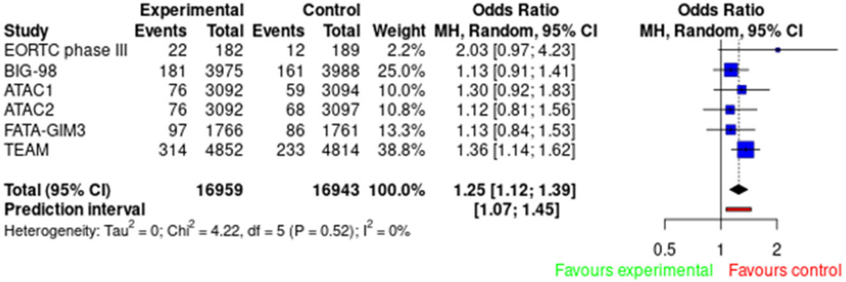

Supplement: Supplementary file 1 [file jcm-11-03133-s001.zip › Supplementary Material S2.pdf]
