# Supplementary material for: Aromatase Inhibitors and Risk of Metabolic and Cardiovascular Adverse Effects in Breast Cancer Patients—A Systematic Review and Meta-Analysis
Source: J Clin Med. 2022 May 31;11(11):3133. doi: 10.3390/jcm11113133 (PMC9181297; doi:10.3390/jcm11113133)
Supplement: Supplementary file 1 [file jcm-11-03133-s001.zip › Supplementary Table S1.pdf]

Supplementary Table S1. Search results in each query - cardiovascular and metabolic adverse events in postmenopausal women with breast cancer receiving aromatase inhibitors (on 27 Oct 2020)

		Embase + Medline
1	'breast tumor':ab,ti OR 'breast tumor'/exp OR ‘breast tumor’	24 363
2	'aromatase inhibitor':ab,ti OR 'aromatase inhibitor'/exp OR 'anastrozole'/exp OR 'arimidex'/exp OR 'letrozole'/exp OR 'femara'/exp OR 'exemestane'/exp OR 'aromasin'/exp	3 008
3	'cardiovascular disease'/exp OR 'ischemic heart disease'/exp OR 'heart infarction'/exp OR 'cerebrovascular accident'/exp OR 'body weight'/exp OR 'obesity'/exp OR 'diabetes mellitus'/exp OR 'dyslipidemia'/exp OR 'glucose intolerance'/exp OR 'insulin resistance'/exp OR 'hyperglycemia'/exp OR 'hypercholesterolemia'/exp OR 'hypertriglyceridemia'/exp OR 'metabolic syndrome X'/exp	264 359
5	#1 AND #2 AND #3	629
