# Supplementary material for: Aromatase Inhibitors and Risk of Metabolic and Cardiovascular Adverse Effects in Breast Cancer Patients—A Systematic Review and Meta-Analysis
Source: J Clin Med. 2022 May 31;11(11):3133. doi: 10.3390/jcm11113133 (PMC9181297; doi:10.3390/jcm11113133)
Supplement: Supplementary file 1 [file jcm-11-03133-s001.zip › Supplementary Table S2.pdf]

Trial	Selection bias		Performance bias	Detection bias	Attrition bias	Reporting bias	Other bias
	Random Sequence Generation	Allocation Concealment	Blinding of participants	Blinding of outcome	Incomplete Outcome	Selective outcome	
**FATA-GIM3 [13]**	-	+	+	+	-	-	?
**SUCCESS C [14]**	?	?	+	+	?	?	-
**FACE [15]**	-	+	+	+	-	-	-
**Iwata et al, 2013 [16]**	-	?	-	-	-	-	?
**MA.27 [17]**	-	+	+	+	-	-	-
**PROACT [18]**	-	?	-	?	-	-	-
**N-SAS BC04 [19]**	?	?	+	+	-	-	-
**TEAM [20]**	-	-	+	+	-	-	-
**N-SAS BC03 [21]**	?	?	+	+	-	-	-
**EORTC, phase III [22]**	-	+	+	+	-	-	-
**ARNO-95 [23]**	-	-	+	+	-	-	-
**IES [24]**	-	-	-	-	-	-	-
**ITA [25]**	?	?	?	?	-	-	-
**BIG-98 [26]**	-	-	-	-	-	-	-
**MA.17 [27]**	?	?	-	-	-	-	-
**EORTC, phase II [28]**	-	+	+	+	-	-	-
**ATAC [29]**	-	-	-	-	-	-	-
**TARGET [30]**	-	?	-	-	-	-	-
**Nabholtz et al, 2000 [31]**	-	-	-	-	?	?	-
**NRG Oncology/NSABP B-42 [32]**	-	-	-	?	?	-	-
**ANZ 0501 LATER [33]**	-	+	+	+	-	-	-

Supplementary Table S2. Quality assessment of included studies. Legend: low risk of bias (-), high risk of bias (+), unclear risk of bias (?)
